# Glycosylation Focuses Sequence Variation in the Influenza A Virus H1 Hemagglutinin Globular Domain

**DOI:** 10.1371/journal.ppat.1001211

**Published:** 2010-11-24

**Authors:** Suman R. Das, Pere Puigbò, Scott E. Hensley, Darrell E. Hurt, Jack R. Bennink, Jonathan W. Yewdell

**Affiliations:** 1 Laboratory of Viral Diseases, NIAID, Bethesda, Maryland, United States of America; 2 National Center for Biotechnology Information, Bethesda, Maryland, United States of America; 3 Computational Biology Bioinformatics and Computational Biosciences Branch (BCBB), NIAID, Bethesda, Maryland, United States of America; Centro de Biología Molecular Severo Ochoa (CSIC-UAM), Spain

## Abstract

Antigenic drift in the influenza A virus hemagglutinin (HA) is responsible for seasonal reformulation of influenza vaccines. Here, we address an important and largely overlooked issue in antigenic drift: how does the number and location of glycosylation sites affect HA evolution in man? We analyzed the glycosylation status of all full-length H1 subtype HA sequences available in the NCBI influenza database. We devised the “flow index” (FI), a simple algorithm that calculates the tendency for viruses to gain or lose consensus glycosylation sites. The FI predicts the predominance of glycosylation states among existing strains. Our analyses show that while the number of glycosylation sites in the HA globular domain does not influence the overall magnitude of variation in defined antigenic regions, variation focuses on those regions unshielded by glycosylation. This supports the conclusion that glycosylation generally shields HA from antibody-mediated neutralization, and implies that fitness costs in accommodating oligosaccharides limit virus escape via HA hyperglycosylation.

## Author Summary

Influenza A virus is highly susceptible to neutralizing antibodies specific for the viral hemagglutinin glycoprotein (HA), and is easily controlled by standard vaccines. Influenza A virus remains an important human pathogen, however, due to its ability to rapidly evade antibody responses. This process, termed antigenic drift, is due to the accumulation of amino acid substitutions that modify HA antigenic sites recognized by neutralizing antibodies. In this study, we perform bioinformatic analysis on thousands of influenza A virus isolates to better understand the influence of N-linked glycosylation on antigenic drift. HA from human IAV isolates can accommodate up to 6 oligosaccharides in its globular domain. We show that for H1, H2, and to a somewhat less extent H3, HAs, the number of glycosylation sites in the globular domain does not greatly modify the total degree of variation in antigenic sites, but rather focuses variation on sites whose access to antibodies is unaffected by glycosylation. Our findings imply that glycosylation protects HA from antibody neutralization, but functional impairment limits the number of oligosaccharides that HA can accommodate.

## Introduction

The influenza A virus (IAV) hemagglutinin (HA) is a homotrimeric glycoprotein that initiates infection by attaching virus to host cell sialic acids and mediating fusion of viral and endosomal membranes [1]. HA consists of a fibrous stem inserted into the viral membrane supporting a globular domain containing three sialic acid binding sites (one per monomer). Trimerization of nascent HA is necessary for HA folding and export from the early secretory pathway [2], [3], [4]. Nearly all antibodies (Abs) that neutralize viral infectivity (“neutralizing antibodies”) recognize epitopes in the globular domain. Most Abs neutralize infection by sterically blocking access of sialic acid receptors to the HA [5], [6].

Neutralizing Abs are the principal selective force driving HA evolution in man. The rapid emergence of mutants that escape Ab neutralization is termed “antigenic drift”, and has prevented effective long-term vaccination against IAV. Based on locating single amino acid substitutions that enable escape from neutralization with monoclonal Abs (mAbs), physically distinct regions have been defined on the globular domains of H1 (Sa, Sb, Ca, Cb) and H3 (A, B, C, D, E) subtype HAs [7], [8], [9]. We term the region of HA containing these sites, consisting of residues 58–272, the globular domain. Differences in the location of the antigenic sites in the globular domain correlate with the differential location of consensus N-linked oligosaccharide attachment sites in the H1 (PR8) *vs.* H3 (HK) HAs used for antigenic analysis [9], [10].

This raises the important question of the influence of HA glycosylation on antigenic drift. Other viral glycoproteins (*e.g.* HIV gp160) mask potential antigenic sites by hyperglycosylation [11], [12]. Addition of glycans to the globular domain has been directly shown to block neutralization of HA by monoclonal and polyclonal Abs [13]. Why doesn't IAV employ this strategy to a greater extent? A potential clue comes from the distinct evolution of H3 *vs.* H1 HAs in humans. Despite circulating for far less time in humans (41 years), H3 viruses have accumulated approximately twice as many glycosylation sites in the globular domain than H1 subtype viruses (circulating for ∼70 years- 1918–1957, 1977-present) [14], [15], [16], [17]. This is consistent with the idea that there are distinct fitness costs to glycosylation that vary among HA subtypes [13], [18], [19], [20].

Despite the potential importance of HA glycosylation in IAV evolution, there is a paucity of bioinformatics analysis of the large number of sequences accumulating in data banks. Here, we provide bioinformatics evidence that supports a critical role for glycosylation in focusing antigenic variation on non-glycosylated regions of the HA globular domain.

## Results

### Distribution of N-glycosylation sites in HA sequences

**Figure 1:**
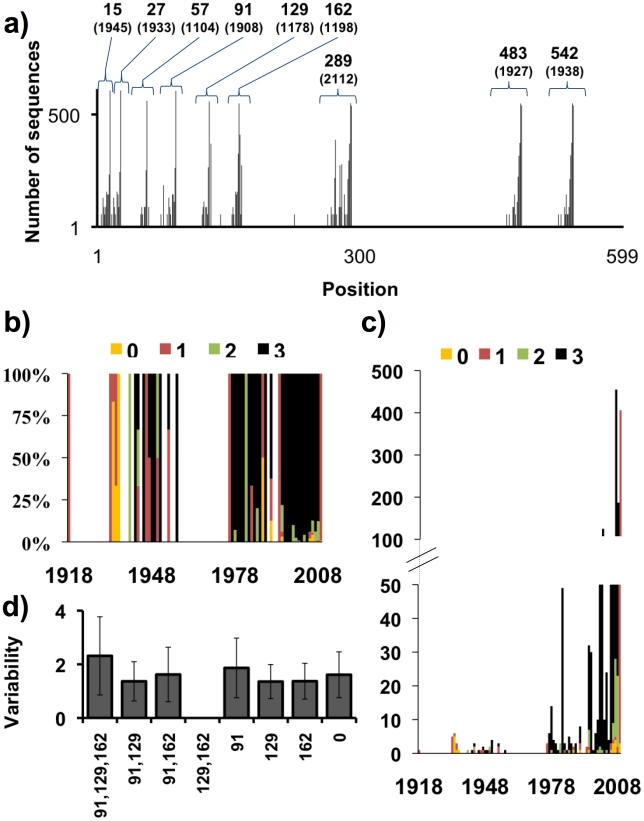
Distributions of the number and variability of glycosylation sites. a) Distribution of glycosylation sites in HA sequences of H1N1 viruses. Each bar corresponds to the number of sequences with a glycosylation site at that position. Numbers on the top of the bars show the positions that tend to be more glycosylated. b) Distribution by years of the percentage of sequences that have 1, 2 or 3 glycosylation sites at the globular domain of HA (percentage). c) Distribution by years of the percentage of sequences that have 1, 2 or 3 glycosylation sites at the globular domain of HA (absolute values). d) Mean amino acids variability (quantitated by counting the number of different amino acids found at each position) +/− standard deviation in sequences with three glycosylation sites at the globular domain at positions 91,129,162, combinations of glycosylation sites of these three positions (there are no sequences with the combination of glycosylation sites at position 129 and 162), a single glycosylation or no glycosylation sites.

We analyzed 1907 full-length H1 HA sequences from human, swine or avian viruses downloaded from the NCBI influenza virus resource. NetNGlyc prediction of glycosylation sites (Asn-Xaa-Ser/Thr, where Xaa is any amino acid except Pro) in the globular domain reveals the non-random distribution of probable glycosylation sites at nine locations (Figure 1a). With few exceptions, glycosylation sites are located within 5 residues on either side of a consensus site. Consequently, for further analysis we defined conserved glycosylation sites within an 11-residue sequence centered on the consensus site.

**Figure 2:**
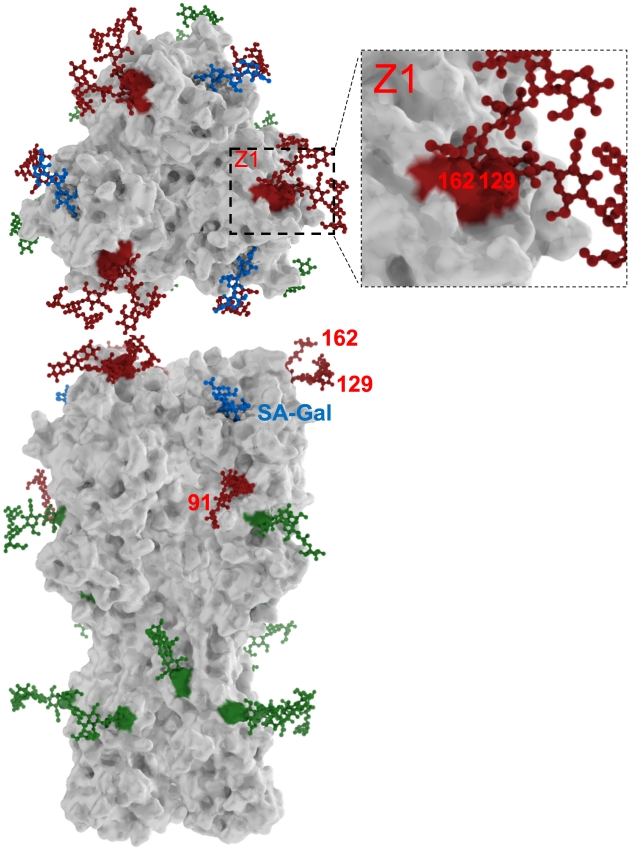
H1N1 HA glycosylation sites. Three-dimensional model of HA as a solid surface viewed from the top and side of the trimeric molecule. Receptor sialic acid oligosaccharides associated with HA are shown in blue. Glycosylation sites are highlighted in green (conserved sites) or red and decorated with complex sugar moieties. Patterns of glycosylation at positions 91, 129 and 162 (red) are important in neutralization. The proximity of residues 129 and 162 clearly limits simultaneous glycosylation at these positions, since steric interference between the oligosaccharides would interfere with folding (zoomed region Z1).

Consistent with previous findings that efficient HA folding and assembly requires glycosylation at conserved sites, glycosylation sites at or near residues 15, 26, 289, 483, and 542 occur in virtually all HAs [4], [20], [26], [27], [28], [29], [30] (note that throughout the manuscript we use the H3 HA numbering system). These sites are located in the stem region of the HA (rendered in green in Figure 2) and may be conserved due to proper association with glycan binding-ER chaperones that facilitate HA folding and assembly [30]. The distribution of glycosylation sites in the H1 globular domain is variable, and is distributed among three regions centered on residues 91, 129 and 162 are rendered in red (Figure 2). For further analysis, we chose ±5 amino acids on each side of the conserved glycosylation sites to define glycosylation regions. It is well documented for H2 HA that addition of consensus glycosylation sites at these regions results in the predicted glycosylation, as determined by mobility shifts in SDS-PAGE [13].

Due to their potential influence on antigenic drift, we focused our attention on the glycosylation sites in the H1 HA globular domain, which center on residues 91, 129, and 162. We temporally analyzed the presence of glycosylation sites in viruses isolated from 1918 to present. Though this analysis is hindered by the limited number of sequences available until 1995, two trends are apparent: an increase in glycosylation sites from zero/one as HA evolved from the 1918 strain to three sites typical for contemporary H1N1 viruses, and an abrupt reintroduction of a single glycosylation site with the appearance of SOIV in 2009 (Figure 1b, c). With three glycosylation sites, there are eight permutations of glycosylation status, all three (1), 2 of 3 (3), one of three (3), and none (1) (Figure 1d). Our analysis revealed the complete absence of HAs with glycosylation sites at positions 129 and 162. Interestingly, these sites are essentially adjacent in the 3-dimensional structure. Thus, it is not surprising that simultaneous glycosylation would have deleterious effects on HA folding, providing strong negative selection; what is more surprising that selection against the two sites is alleviated by a glycosylation site at residue 91, which is located further down the HA trimer (Figure 2). Since glycosylation occurs co-translationally, glycosylation at 91 would precede glycosylation at 129/162, and could limit the extent to which 129 and 162 are simultaneously glycosylated, accounting for its ability to modulate negative selection against adjacent sites. Alternatively, the absence of 129 162 dual glycosylation isolates may relate to historical evolution factors.

### Affect of glycosylation on HA evolution

**Figure 3:**
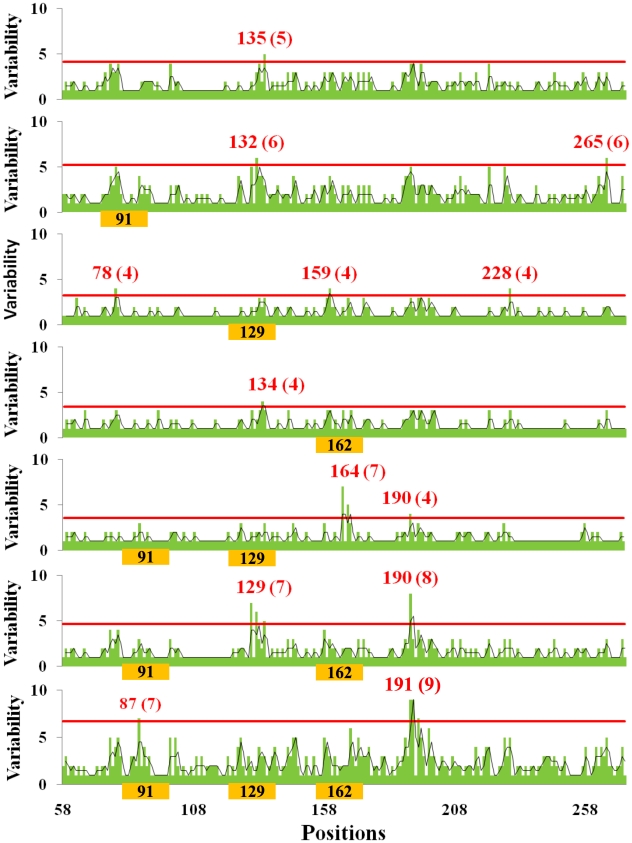
Relationship between amino acid variability and presence of glycosylation sites in H1 globular domain. The plots correspond to H1 HA with the glycosylation sites indicated on the X-axis. Sequences with 2 glycosylation sites in regions 129 and 162 were not found. Green bars plot the number of amino acids present at each position in the group of isolates with specific number of oligosaccharide sites indicated, black lines are the running average of two neighboring positions. The positions and the number of different amino acid residues in each hypervariable region (in parentheses) are shown in red, i.e., those regions that have a variability of 3 standard deviations over the mean value (red line). Number of isolates available for each glycosylation stat: zero sites, 21 isolates; 1 site, position 91, 420 isolates; position 129, 10 isolates; position 162, 4 isolates; 2 sites, positions 91, 129, 34 isolates; 91, 162, 33 isolates; 3 sites, 1118 isolates.

Does glycosylation focus drift on selected antigenic sites? We correlated the location of glycosylation sites with the variability at individual residues in the globular domain (Figure 3). This revealed that glycosylation alters the focus of sequence variation. In HAs lacking glycosylation sites in the antigenic domain, variability peaks near residue 135. Acquisition of a glycosylation site in the same region (129) results in reduction of variability in that region, and increase in variability at residues 78, 159, and 228, which represent the Cb, Sb, and Ca antigenic sites. Acquisition of two glycosylation sites at 91 and 129 now focuses variation at residues 165 and 190. With all three glycosylation sites utilized, variation is now focused around residues 190 and 191. Interestingly, positions 190 and 228 greatly influence HA receptor specificity for α-2,3 *vs.* α-2,6 of the sialic residue [31], [32].

**Figure 4:**
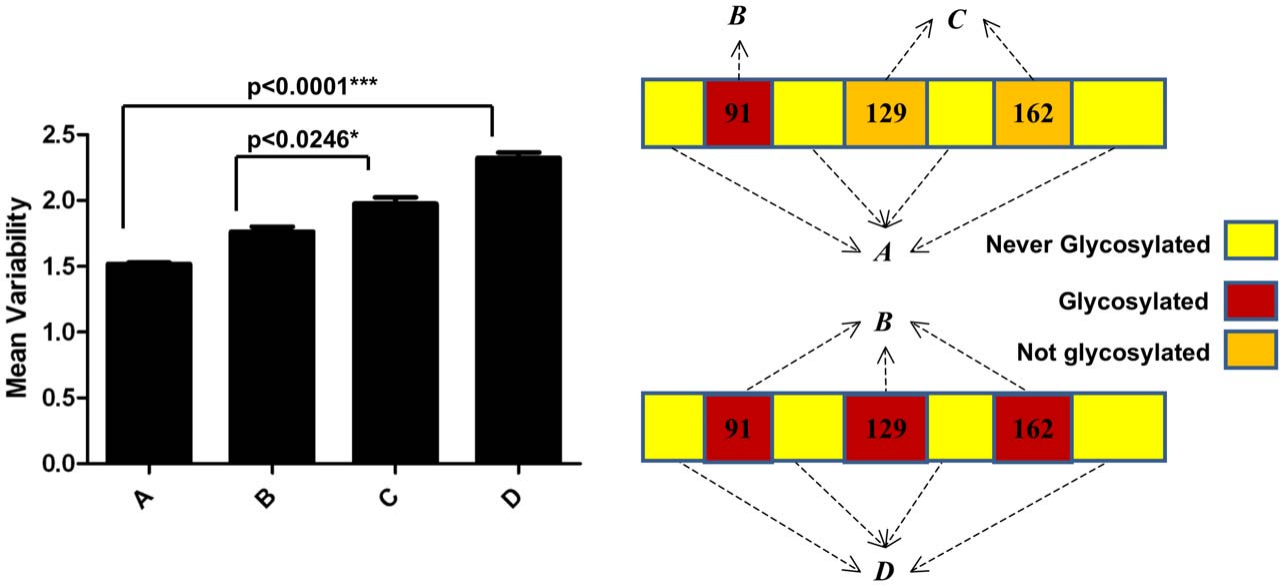
Mean values of variability of H1 globular domain. Mean values of variability at A) non-glycosylated regions of HA globular domain; B) glycosylation sites that are glycosylation competent (i.e. possess consensus glycosylation sites); C) regions that are glycosylated in HA but lack glycosylation sequences; D) non-glycosylated regions of HA from sequences with 3 glycosylation sequences. To reconstruct this plot, glycosylated regions considered positions 91,129 and 162 +/−3 amino acids. Confidence intervals estimated by bootstrap of 500 replicates [44]. Schematic representations of the regions used to calculate mean values of A, B, C and D are shown on right.

Each combination of glycosylation sites generates a similar pattern: glycosylation minimizes variation around its own site while focusing variation onto non-glycosylated sites. The statistical significance of this conclusion is shown in Figure 4. A simple interpretation for this finding is that oligosaccharides shield antigenic regions from Ab neutralization, shifting variation to unshielded sites. This is consistent with the observation that viruses cluster in the PCA plot based on a common number of glycosylation sites in the globular domain and not year of collection (Figure S2), which demonstrates that they are highly homologous in the hypervariable regions of the globular domain.

If glycosylation can affect the pattern of antigenic variation, is there a correlation between number of antigenic domain glycosylation sites and antigenic variation? There is no clear relationship between the number of sites and the overall variability of amino acids between the residues that comprise the globular domain (58–272) (Figure 1d), indicating that the overall extent of glycosylation does not globally limit variation in antigenic regions.

**Figure 5:**
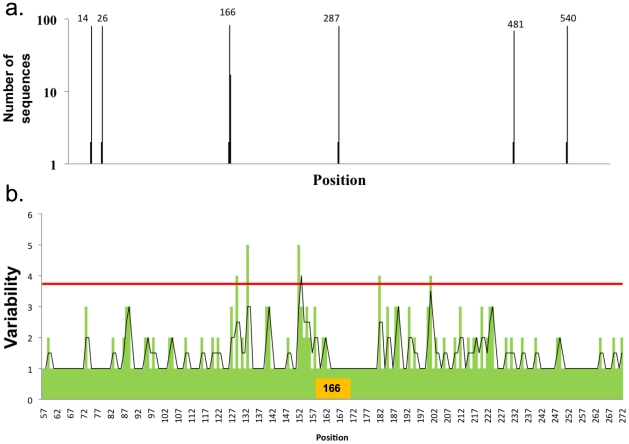
Distribution of glycosylation sites in H2N2 viruses and influence on HA variability. a) Distribution of glycosylation sites in H2N2 viruses as in Figure 1A, 83 full-length sequences were used in this analysis. b) Variability in the globular domain of H2N2 viruses, as in Figure 3.

We extended this approach to H2N2 and H3N2 HA sequences. H2N2 viruses possess a single glycosylation site in the globular domain, located at position 166 (Figure 5a). Consistent with the H1 data, analysis of H2N2 viruses showed limited variation near the sole glycosylation site at position 166 (Figure 5b).

**Figure 6:**
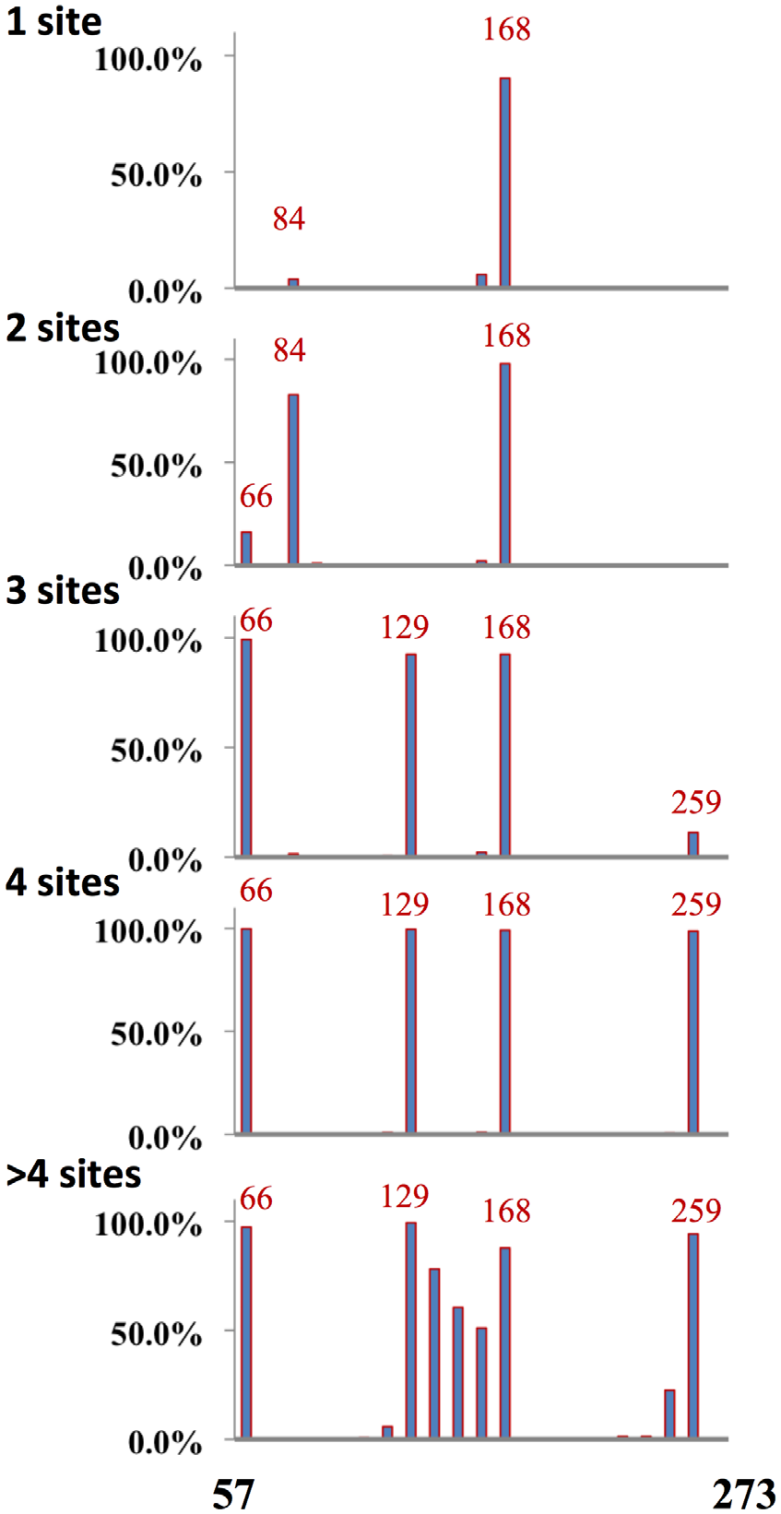
Distribution of glycosylation sites H3N2 viruses. H3N2 viruses were binned according to the number of glycosylation sites in the globular domain as indicated. Plotted is the percentage of viruses with glycosylation sites in the position designated. A total of 2791 H3N2 full-length sequences were used in this analysis.

H3N2 viruses have up to six glycosylation sites on the globular domain of HA (Figure 6). When we sorted H3 sequences based on number of glycosylation sites, we found a distinct trend compared to H1 HA in acquiring glycosylation sites. Position 168 is the most conserved position glycosylation in H3 sequences. When there is one glycosylation, it's nearly invariably at position 168 (with a few isolates with lone glycosylation at position 84). Double glycosylation is dominated by the 84, 168 pairing. Remarkably, triple gycosylation is dominated by 168 with two novel sites: 66 and 129. Adding glycosylation at residue 259 uniformly attains four-site glycosylation. Adding sites between residues 129 and 168 achieve higher order glycosylation.

**Figure 7:**
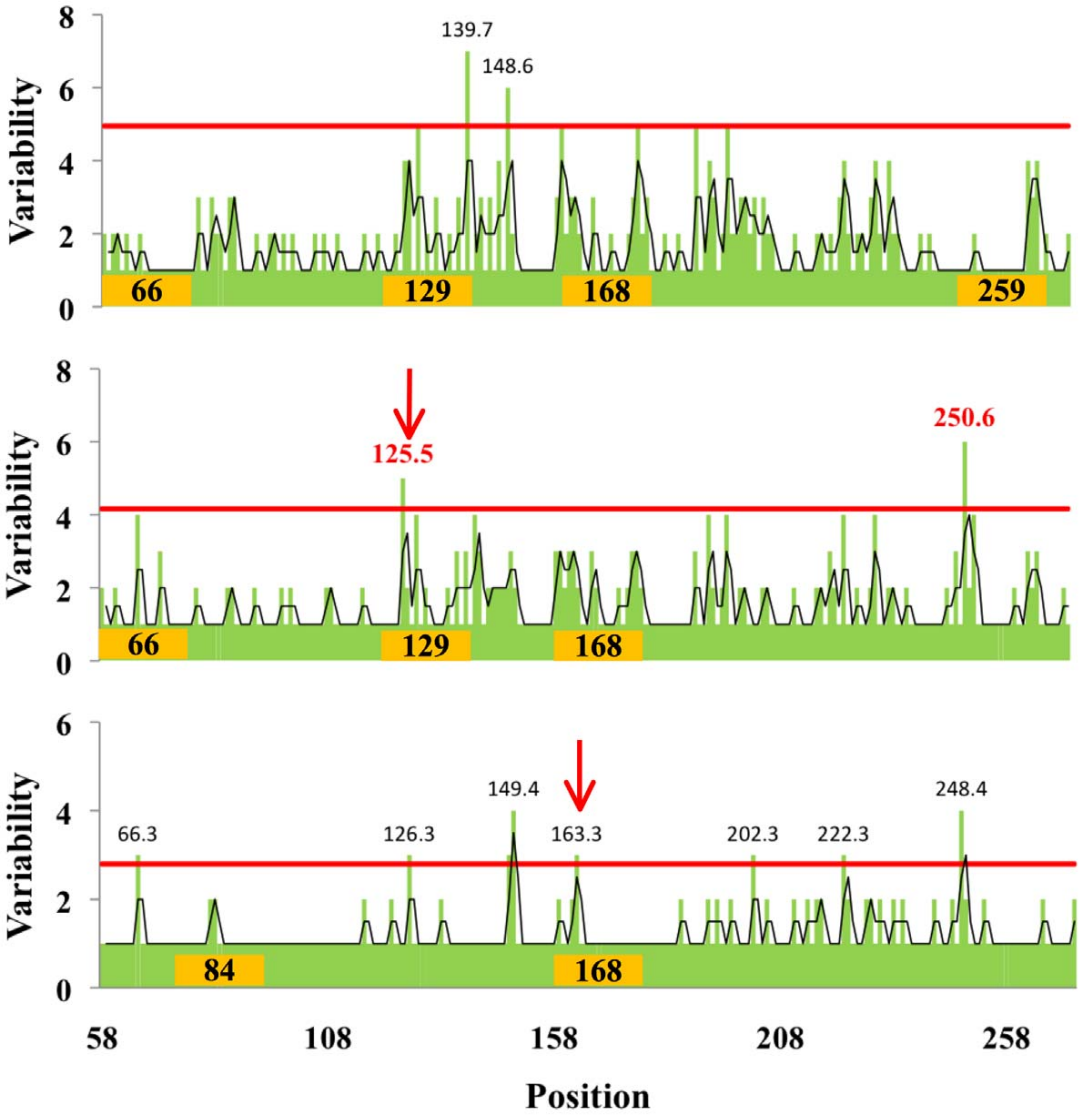
Relationship between amino acid variability and presence of glycosylation sites in H3 globular domain. Variability in the globular domain of H3N2 viruses as in Figure 3. Arrows point to glycosylation sites that do not limit variability in the adjacent residues.

We next examined the correlation between the location glycosylation site and regions of variability for H3 viruses with 2 to 4 sites in the globular domain (Figure 7). Analysis of other glycoforms was compromised by either paucity of isolates in a group or by the complexity of glycosylation pattern. Although there was a reasonable correlation between the presence of a glycosylation site and absence of variation in the residues surrounding the site, this relationship was less robust than for the H1 and H2 HAs (as indicated by the arrows pointing to exceptions).

### Predicting oligosaccharide evolution

**Figure 8:**
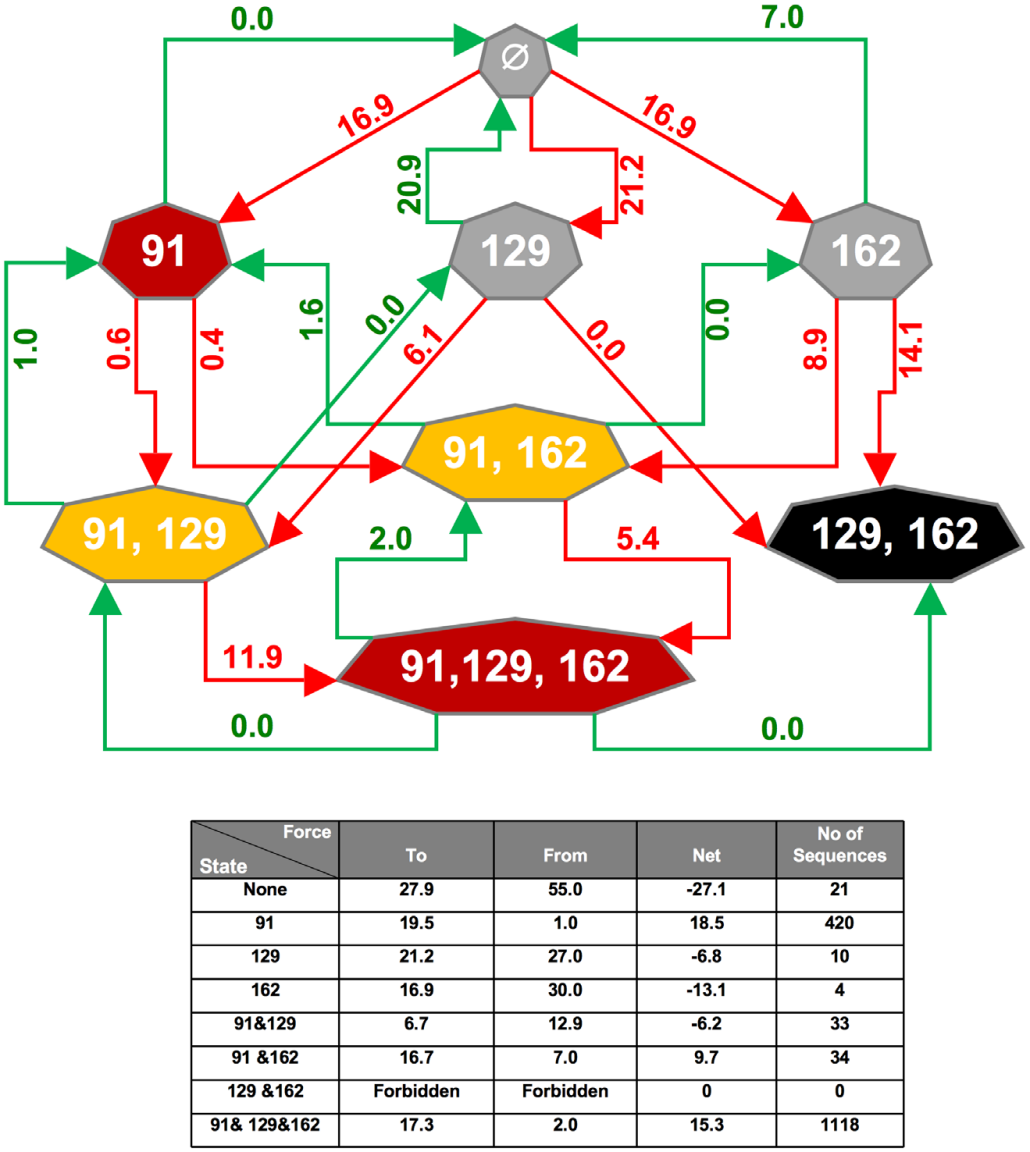
Schematic representation of the flow among the different states of the virus generated by the Flow Index. Each heptagon represents a glycosylation state of H1 HAs from human viruses isolated until mid-2009 (i.e. no Swine origin HAs are represented). Based on the number of isolates with each glycosylation pattern (show in the Table below as number of sequences), we binned viruses into optimal (red), sub-optimal states (yellow), transitional (grey) and lone lethal state (black). The different states are connected by red arrows to indicate an increase in the number of glycosylation from the pre-state to the post-state or green arrows to indicate a decrease in the number of glycosylation sites. Values of arrows indicate the Flow Index (FI), i.e., the tendency of going from one pre-state to a post-state. Data for figure are provided in Table S1. The net FI acting on each state is given by the sum of the forces as calculated in the Table below, note that this correlates well with the number of isolates in a given state.

Is it possible to predict the tendency of acquisition of glycosylation sites (losses as well as gains) as a function of likelihood of mutation of codons present in the glycosylation regions? We devised a simple algorithm, termed the flow index (FI) to model glycan site evolution based on the sequences present in the glycosylation regions of H1N1 viruses with a given oligosaccharide status. The tendency of mutating to (green arrow) or from (red arrow) a given glycosylation state is assigned is shown in Figure 8. Summing the probabilities to and from a given state provides a measure of the probability of remaining in that state (Table S1).

Despite its simplicity, this algorithm reasonably accurately reflects the prevalence of glycoforms among the H1 isolates in the database. The most notable exception is the 129+162 glycoform, which is not represented by any isolate despite having a facile mutational path. As noted above, this may be due to the proximity of these residues in the folded structure, which may interfere with folding of the globular domain (Figure 2). This exception points to the contribution of functional selection in the prevalence of glycoforms.

Unfortunately, we could not calculate a FI for H2 or H3 HA evolution due to either low number of sequences of viruses in a given group or the complexity of the glycosylation patterns.

### Analysis of SOIV evolution

The recent introduction of SOIV into the human population offers a unique opportunity to study IAV evolution in humans at high resolution in real time [33], [34], [35]. Nearly all of the 212 unique SOIV isolates downloaded on October 12^th^ 2009 possess oligosaccharide site at position 91. How does the pattern of variation of SOIVs compare to human H1 isolates or classic Swine isolates that also possess a single oligosaccharide site at position 91?

**Figure 9:**
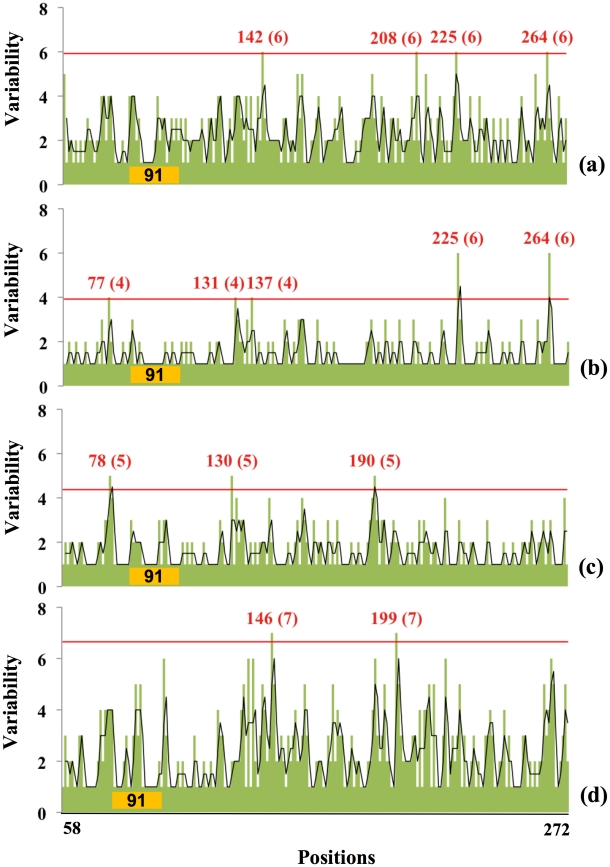
Relationship between amino acid variability and presence of glycosylation sites in Swine H1 globular domain. The plots correspond to a) Swine-origin 2009 H1N1 HA sequences (31^st^ March, 2010) which has 1 glycosylation site in region 91; b) Swine-origin 2009 H1N1 HA sequences (12^th^ October, 2009) which has 1 glycosylation site in region 91; c) Human virus HAs from 1918–2008 with 1 glycosylation site in region 91; d) Swine virus HA sequences from 1918–2008 with 1 glycosylation site in region 91. The positions and the number of possible amino acids of hyper-variable regions are shown as in Figure 3.

As seen in Figure 9, despite their limited time in humans, SOIVs demonstrate a remarkable amount of variation, peaking around positions 225 and 264, with other hot spots at residues 77 and 135 (Figure 9b). This pattern differs from human H1N1 isolated from 1918 to present (Figure 9c), which show far less variation at residues 225 and 264 regions while focusing variation near 77, 135 and 190 regions. Classic swine viruses (Figure 9d) show a different pattern of variation, focused at residues 147 and 200 (note that the data shown in Figure 3b include all isolates with a single glycosylation site at position 91).

We again downloaded available unique full-length SOIV sequences on March 31^st^ to examine variability over the course of six months (Figure 9a). Apart from variability at position 225 and 264, there is a higher variability near position 142 (Sb-site) that was absent in October.

Can we predict the evolution of SOIV glycoforms using the FI? FI of the October sequences for loss of oligosaccharide at 91 or gain at 129 or 162 is each calculated to be zero, *i.e.* two amino substitutions are required to insert a glycan at these regions. Indeed, the sequences downloaded on March 2010 showed a strong tendency to maintain glycosylation at position 91, though a few isolates lose glycosylation at 91 (10 isolates) or gain glycosylation at 162 (5 isolates) [36]. We therefore predict that a single glycosylation site at position 91 at the antigenic domain will predominate in SOIV isolates for a prolonged period.

## Discussion

It has been known for more than 70 years that IAV, unlike many viruses, demonstrates significant antigenic variation. The epidemiological significance of antigenic variation in IAV was unmistakable from the failure of the 1947 vaccination campaign [37], [38]. Despite considerable effort and significant gains in understanding HA antigenic structure, much remains to be learned about how drift occurs in humans. The revolution in nucleic acid sequencing technology provides enormous opportunities to better understand drift. Here, we utilize the NCBI influenza resource to examine the relationship between glycosylation in the HA globular domain and antigenic variation.

The ability of oligosaccharides to sterically block antibody binding to HA antigenic sites was clearly established with the original definition of antigenic sites on the HA structure using mAbs [39], [40]. Surprisingly, however, the more global effects of oligosaccharides on HA evolution have been examined in only a few publications [17], [41], [42]. We detect a clear influence of oligosaccharides in directing the focus of variation to the established neutralizing antibody binding sites on the H1 and H2 HAs. We also find a similar pattern among H3 HAs with 2–4 globular domain glycosylation sites, but note exceptions to the relationship (arrows in Figure 6), that might contribute to the finding of a prior bioinformatics analysis of H3 isolates that failed to detect a relationship between glycosylation and the locus of variability [41]. This potential difference in glycosylation in shaping HA evolution might be related to a major difference in H1 vs. H3 HA evolution: while H3 remained in human populations constantly from its introduction in 1968 until the present time, after the complete replacement of H1 in 1957 by H2 viruses, it re-appeared in 1977 in the form of the 1950 virus, almost certainly as a result of a re-introduction from a laboratory sample.

While there is a tendency towards adding oligosaccharides to the H1 HA with time, the process is slower than might be expected. H1 viruses have circulated in humans for at least 80 years in the period between 1918 and present time, yet only possess 3 globular domain glycosylation sites while H3 HAs have up to six glycosylation sites in the globular domain.

It is important to note that we have not experimentally established that antibody pressure is responsible for the influence of oligosaccharides on variation in the globular domain. Although it seems less likely, it is possible that oligosaccharides influence HA evolution by modulating the mutation space of globular domain residues.

We show that the sequence space in the regions of the globular domain where oligosaccharides can be accommodated appears to play a surprisingly robust role (since at most, only two amino acid changes are needed to create a glycosylation site) in influencing the evolutionary acquisition of additional glycosylation sites. Thus, although the FI is hampered by historical biases in the number of isolates collected during the course of IAV evolution in man (and by alterations in glycosylation that accompany adaptation to growth in eggs or cultured cells [43]), it nonetheless is able to predict the prevalence of HA glycoforms in H1N1 isolates. That the FI is a less than perfect prognosticator is expected, since sequence space does not completely account for oligosaccharide evolution. A critical missing factor is the fitness of the various glycoforms, both in terms of viral replication in the human host, and also the ability of virus to evade neutralizing antibodies. Oligosaccharides are well known to influence HA function, particularly binding to host cell receptors and of course, in shielding HA from Ab mediated neutralization.

Indeed, the major point of this work is that oligosaccharides influence HA evolution in antigenic regions. Notably, while the number of oligosaccharides in the globular domain has little gross effect on the overall variation (Figure 1d), it focuses variation on uncovered antigenic epitopes. This supports the idea that glycosylation is an effective strategy for deflecting neutralizing Abs. Why then, doesn't HA simply cover itself with oligosaccharides?

The likely answer is that HA simply can't block all neutralization sites with oligosaccharides and maintain its function. This may be a more difficult evolutionary task than it appears at first glance, since HIV gp160 is the exception among viral receptor proteins rather than the rule. Perhaps there are yet to be defined host molecules that recognize hyperglycosylated proteins to limit this strategy.

## Materials and Methods

### Source of sequences

A first set of 4781 full length HA sequences (full-length sequences from all hosts and geographic origins) were downloaded on June 26^th^, 2009 from the influenza virus resource at the NCBI (http://www.ncbi.nlm.nih.gov/genomes/FLU). These include 1907 H1N1, 83 H2N2 and 2791 H3N2 sequences. A second set of 212 swine origin influenza virus (SOIV) H1N1 HA sequences) was downloaded on 12^th^ October 2009, followed by a third set of 1339 full-length SOIV sequence was downloaded on March 31^st^ 2010.

### Prediction of N-glycosylation sites

The NetNGlyc 1.0 web-server (http://www.cbs.dtu.dk/services/NetNGlyc) was used to predict N-Glycosylation sites (Asn-Xaa-Ser/Thr, where Xaa is any amino acid except Pro) of all HA sequences; a positive was scored when the jury returned a “+” score. According to NetNGlcy, 76% of positive scored sequons are modified by N-Glycans, with a bias towards Thr containing sequons [21], [22], [23], [24].

### Multiple sequence alignment

All HA sequences of H1N1 were aligned in a single common alignment using the program Muscle [25] with default parameters.

### Principal Components Analysis of the amino acid composition

The amino acids composition of the sequences was used to perform a multivariate analysis called Principal Components Analysis (PCA). The PCA analysis of the amino acids composition was performed using the prcomp function of the R package (http://www.r-project.org). This analysis performs a decomposition of the variables, e.g. the abundance of each amino acid (20 variables), into each principal component. The first two components of the PCA, showed in the plots 1 and 2, preserve 59% of the total variability (Figure S2).

### Amino acid variability

Amino acid variability was quantitated from position 58 through 272 (globular domain). Figure 3 shows the amount of variability in H1 HA at each position. Variability was quantitated by counting the number of different amino acids found at each position, i.e. a position where all sequences have the same amino acid, the value of variability is 1, while for example a variability value of 7 corresponds to a position that have 7 different possible amino acids. Likewise, variability of H2 HA and some H3 HAs at each position were calculated (Figure 4 and 6). Regions of the H1 HA globular domain 91, 129 and 162+/−5 amino acids were selected to calculate the Flow Index.

### Defining the “Flow Index”

H1 HA sequences were sorted based on their glycosylation status (i.e., Ø; 91; 129; 162; 91,129; 91,162; 91,129,162). Sequences with the glycosylation sites at positions 129 and 162 were not found. The amino acids frequencies in each aligned amino acid position of these regions for each starting group were calculated. Then, using the amino acids frequencies at each position, a set of 10,000 “random” sequences of each group was generated. These “random” sequences, which maintain the amino acids frequencies of the actual sequences, correspond to the initial “pre-state” to run the simulations.

We performed two independent rounds of simulation (flow-charted in Figure S1). Since the tendency of the virus is to maintain its glycosylation status, a change in status rarely occurs in a single round of simulation. The first round (left side Figure S1) uses the amino acids frequencies of each pre-state. Then, choosing a position at random in a glycosylation region, an amino acid substitution based on the amino acid frequencies at the same position is made (*i.e.* random substitution guided by the amino acids frequencies of the pre-state sequence). Using this data set, we enumerated the number of times that single changes in glycosylation site number occurred (gain or loss) per 10,000 iterations, and calculated the Pd_i_, the probability of changing glycosylation status. In the second simulation round (right side, Figure S1), repeated rounds of simulation are performed until a change occurs, resulting in Pd_i→j_ the probability of changing from a pre-state to a post-state that differ by a single glycosylation site (gain or loss). The Flow Index (FI) is defined as the product of the two rounds and provides a measure of the tendency of changing from a pre-state i to a post-state j.
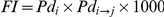
Since the FI is based on the frequency of amino acid of all sequences in the starting group, it is free of constraints imposed by a consensus sequence. In addition, the FI also takes selection into account, since only sequences of viable viruses are used in the simulated mutagenesis.

## Supporting Information

Table S1 Data for FI.
(0.06 MB DOC)

Figure S1 Flow Chart of Flow Index algorithm.
(0.65 MB TIF)

Figure S2 Representation of the first and second components of the Principal Components Analysis (PCA) for H1 viruses. PCA of the amino acids composition of H1N1. The first two components of the PCA account for 59% of the total variability. a) PCA plot by host. Blue diamonds correspond to sequences found in avian viruses, green triangles correspond to sequences found in swine viruses, and red squares correspond to sequences found in human viruses. b) PCA plot by number of glycosylation sites. Red squares correspond to sequences without glycosylation sites, blue diamonds correspond to sequences with 1 glycosylation site, green triangles correspond to sequences with 2 glycosylation sites, purple crosses correspond to sequences with 3 glycosylation sites and light blue stars correspond to sequences with 4 glycosylation sites in the globular domain. c) Distribution of the values of the first component (PC1) by years. Blue bars correspond to sequences found in avian viruses, green bars correspond to sequences found in swine viruses, and red bars correspond to sequences found in human viruses. d) Distribution of the values of the second component (PC2) by years. Blue bars correspond to sequences found in avian viruses, green bars correspond to sequences found in swine viruses, and red bars correspond to sequences found in human viruses.
(1.13 MB TIF)
