# Supplementary material for: Glycosylation Focuses Sequence Variation in the Influenza A Virus H1 Hemagglutinin Globular Domain
Source: PLoS Pathog. 2010 Nov 24;6(11):e1001211. doi: 10.1371/journal.ppat.1001211 (PMC2991263; doi:10.1371/journal.ppat.1001211)
Supplement: Table S1 — Data for FI. (0.06 MB DOC) [file ppat.1001211.s001.doc]

N_Pre-state_= Number of sequences found.

Pe_i_= Probability of being in the same state.

Pd_i_= Probability of going to a different state.

Pd_i->j_= Probability of going to a post-state given a pre-state.

FI = Flow Index = Pd_i_*****Pd_i->j_*****1000

| **States** | **Pre-state** | **N_Pre-state_** | **Pe_i_** | **Pd_i_** | **Post-state** | **Pd_i->j_** | **FI** |
| --- | --- | --- | --- | --- | --- | --- | --- |
| **Lethal** | **129,162** | 0 | - | - | - | - | - |
| **Transitional** | **∅** | 21 | 0.945 | 0.055 | **91** | 0.308 | 16.918 |
|  |  |  |  |  | **129** | 0.385 | 21.181 |
|  |  |  |  |  | **162** | 0.307 | 16.902 |
|  | **129** | 10 | 0.973 | 0.027 | **∅** | 0.775 | 20.933 |
|  |  |  |  |  | **91,129** | 0.225 | 6.067 |
|  |  |  |  |  | **129,162** | 0.000 | 0.000 |
|  | **162** | 4 | 0.970 | 0.030 | **∅** | 0.235 | 7.035 |
|  |  |  |  |  | **91,162** | 0.296 | 8.877 |
|  |  |  |  |  | **129,162** | 0.470 | 14.088 |
| **Sub-optimal** | **91,162** | 33 | 0.993 | 0.007 | **91** | 0.235 | 3.060 |
|  |  |  |  |  | **162** | 0.000 | 0.000 |
|  |  |  |  |  | **91,129,162** | 0.765 | 9.940 |
|  | **91,129** | 34 | 0.987 | 0.013 | **91** | 0.081 | 1.049 |
|  |  |  |  |  | **129** | 0.000 | 0.000 |
|  |  |  |  |  | **91,129,162** | 0.919 | 11.951 |
| **Optimal** | **91** | 420 | 0.999 | 0.001 | **∅** | 0.000 | 0.000 |
|  |  |  |  |  | **91,129** | 0.599 | 0.599 |
|  |  |  |  |  | **91,162** | 0.401 | 0.401 |
|  | **91,129,162** | 1118 | 0.998 | 0.002 | **91,129** | 0.012 | 0.024 |
|  |  |  |  |  | **91,162** | 0.988 | 1.976 |
|  |  |  |  |  | **129,162** | 0.0003 | 0.001 |
