# Supplementary figures and images for: Glycosylation Focuses Sequence Variation in the Influenza A Virus H1 Hemagglutinin Globular Domain
Source: PLoS Pathog. 2010 Nov 24;6(11):e1001211. doi: 10.1371/journal.ppat.1001211 (PMC2991263; doi:10.1371/journal.ppat.1001211)

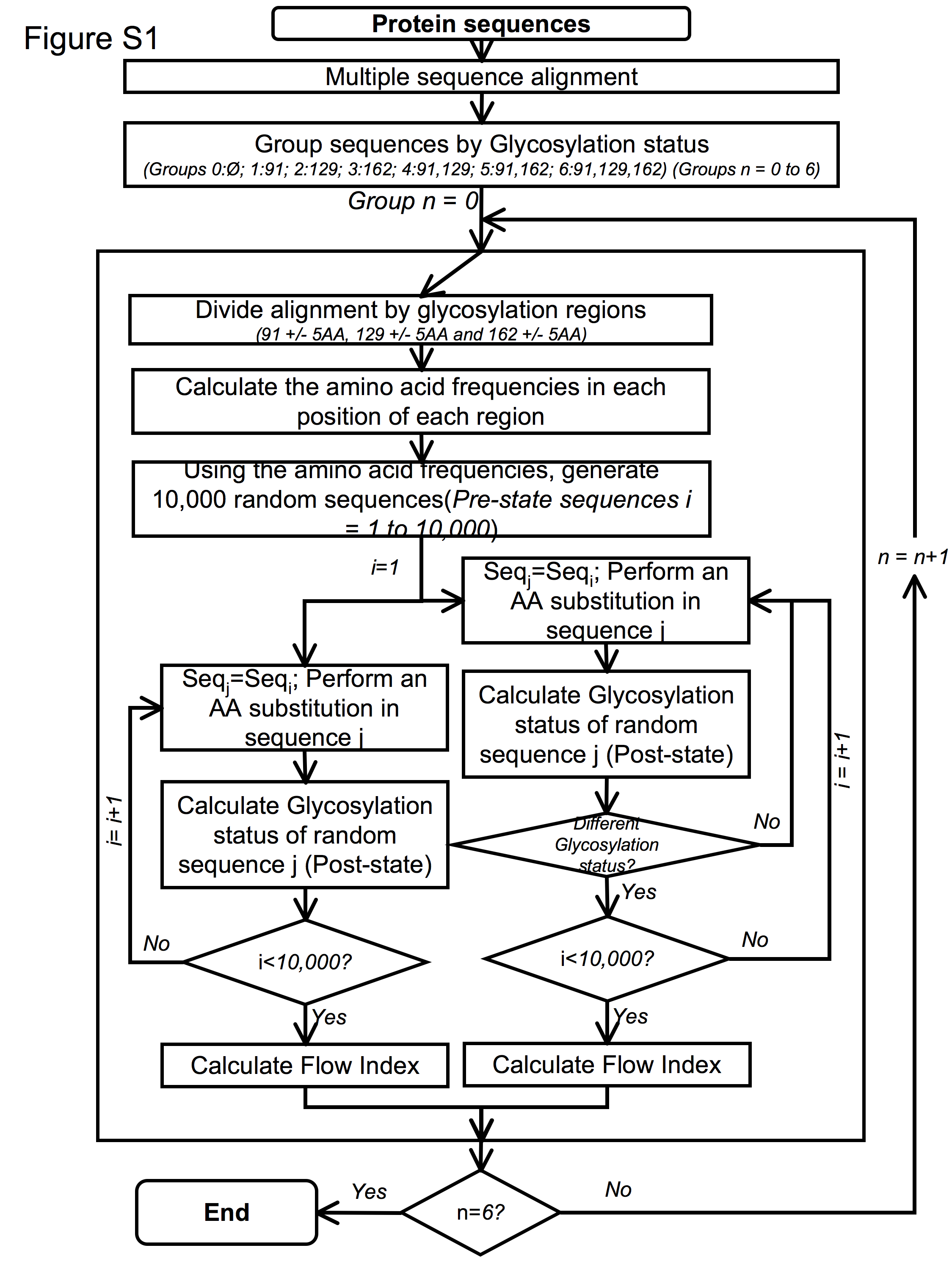

Supplement: Figure S1 — Flow Chart of Flow Index algorithm. (0.65 MB TIF) [file ppat.1001211.s002.tif]

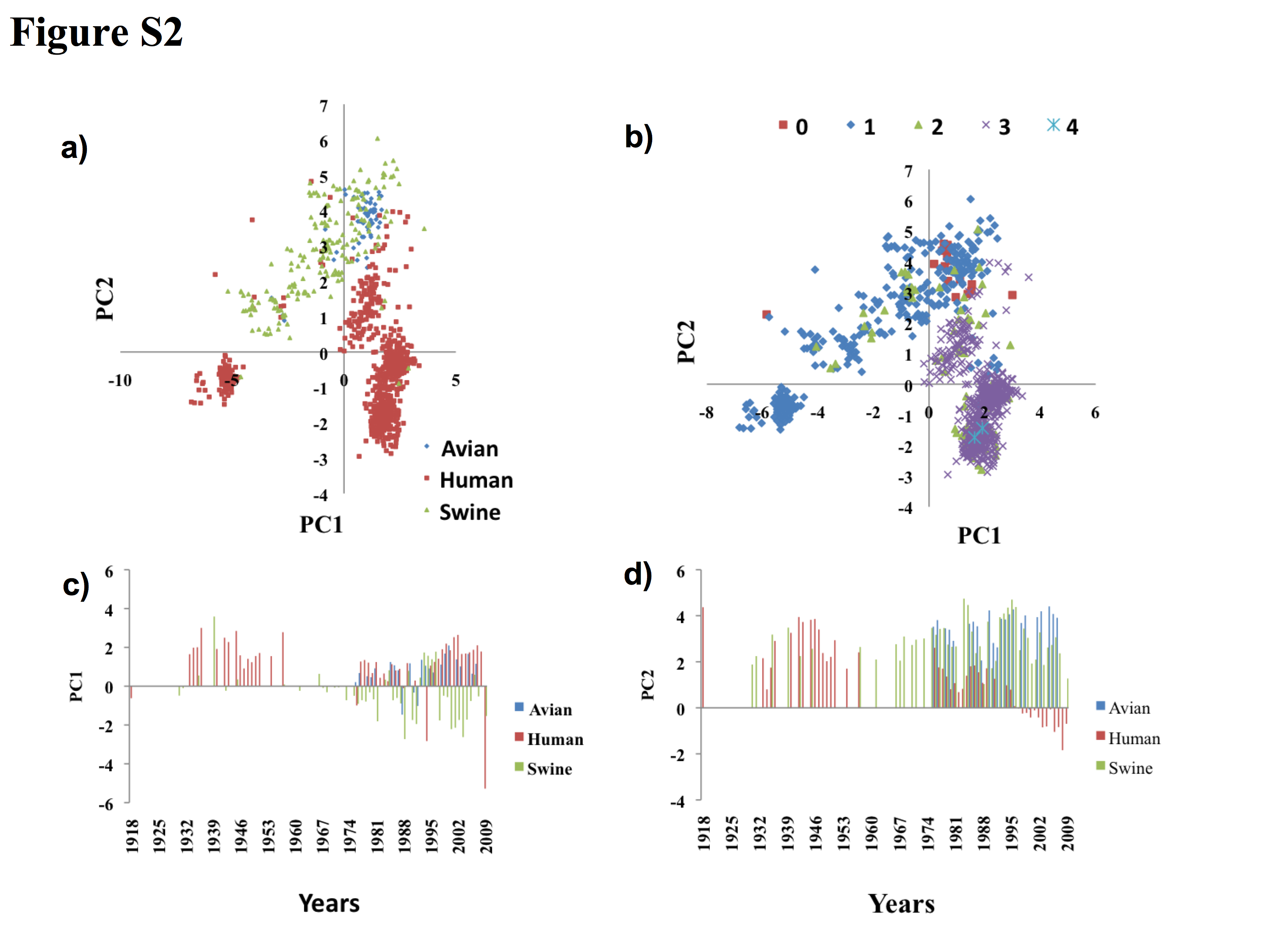

Supplement: Figure S2 — Representation of the first and second components of the Principal Components Analysis (PCA) for H1 viruses. PCA of the amino acids composition of H1N1. The first two components of the PCA account for 59% of the total variability. a) PCA plot by host. Blue diamonds correspond to sequences found in avian viruses, green triangles correspond to sequences found in swine viruses, and red squares correspond to sequences found in human viruses. b) PCA plot by number of glycosylation sites. Red squares correspond to sequences without glycosylation sites, blue diamonds correspond to sequences with 1 glycosylation site, green triangles correspond to sequences with 2 glycosylation sites, purple crosses correspond to sequences with 3 glycosylation sites and light blue stars correspond to sequences with 4 glycosylation sites in the globular domain. c) Distribution of the values of the first component (PC1) by years. Blue bars correspond to sequences found in avian viruses, green bars correspond to sequences found in swine viruses, and red bars correspond to sequences found in human viruses. d) Distribution of the values of the second component (PC2) by years. Blue bars correspond to sequences found in avian viruses, green bars correspond to sequences found in swine viruses, and red bars correspond to sequences found in human viruses. (1.13 MB TIF) [file ppat.1001211.s003.tif]
